# A Peroxisomal Long-Chain Acyl-CoA Synthetase from *Glycine max* Involved in Lipid Degradation

**DOI:** 10.1371/journal.pone.0100144

**Published:** 2014-07-03

**Authors:** Lili Yu, Xiaoli Tan, Bingjun Jiang, Xuegang Sun, Shoulai Gu, Tianfu Han, Wensheng Hou

**Affiliations:** 1 The National Key Facility for Crop Gene Resources and Genetic Improvement (NFCRI), Institute of Crop Sciences, Chinese Academy of Agricultural Sciences, MOA Key Laboratory of Soybean Biology (Beijing), Beijing, China; 2 Institute of Life Sciences, Jiangsu University, Zhenjiang, Jiangsu Province, China; 3 Research Center for Immunology, Department of Immunology, School of Basic Medical Sciences, Xinxiang Medical College, Henan Province, Xinxiang, China; Case Western Reserve University, United States of America

## Abstract

Seed storage oil, in the form of triacylglycerol (TAG), is degraded to provide carbon and energy during germination and early seedling growth by the fatty acid β-oxidation in the peroxisome. Although the pathways for lipid degradation have been uncovered, understanding of the exact involved enzymes in soybean is still limited. Long-chain acyl-CoA synthetase (ACSL) is a critical enzyme that activates free fatty acid released from TAG to form the fatty acyl-CoA. Recent studies have shown the importance of ACSL in lipid degradation and synthesis, but few studies were focused on soybean. In this work, we cloned a *ACSL* gene from soybean and designated it as *GmACSL2*. Sequence analysis revealed that *GmACSL2* encodes a protein of 733 amino acid residues, which is highly homologous to the ones in other higher plants. Complementation test showed that GmACSL2 could restore the growth of an ACS-deficient yeast strain (YB525). Co-expression assay in *Nicotiana benthamiana* indicated that GmACSL2 is located at peroxisome. Expression pattern analysis showed that *GmACSL2* is highly expressed in germinating seedling and strongly induced 1 day after imbibition, which indicate that *GmACSL2* may take part in the seed germination. *GmACSL2* overexpression in yeast and soybean hairy root severely reduces the contents of the lipids and fatty acids, compared with controls in both cells, and enhances the β-oxidation efficiency in yeast. All these results suggest that *GmACSL2* may take part in fatty acid and lipid degradation. In conclusion, peroxisomal *GmACSL2* from *Glycine max* probably be involved in the lipid degradation during seed germination.

## Introduction

Seed germination and maturation depend on the breakdown of stored triacylglycerols (TAGs), which are degraded to provide carbon and energy through the successive operations of the fatty acid oxidation, glyoxylate cycle, partial tricarboxylic acid cycle, and gluconeogenesis [1]. At the beginning of lipid degradation, the main storage lipids in oil bodies are first hydrolyzed by lipases to the free fatty acids. The conversion of these fatty acids to succinate by β-oxidation occurs in peroxisomes, which are single membrane-bound organelles that carry out many essential lipid metabolic reactions. Peroxisome metabolism is crucial to all stages of plant growth, including seed development, germination, general growth, and senescence. Moreover, peroxisome is a major site for β-oxidation metabolism of several of long-chain aliphatic, branched, and aromatic carboxylic acids [2], [3], [4].

Fatty acids released from TAGs must be activated to acyl-CoAs. This reaction is catalyzed by long-chain acyl-CoA synthetase (ACSL, EC 6.2.1.3), which converts free fatty acids into acyl-CoA thioesters as the substrates for β-oxidation. Because free fatty acids are chemically inert, the activation to CoA thioester of fatty acids is essential and accessible to several biochemical pathways [5]. This thioesterification is catalyzed by ACSL in two steps. At the first step, free fatty acid is formed to an acyl-AMP intermediate, called an adenylate, through ATP pyrophosphorolysis. At the second step, the activated carbonyl carbon of the adenylate is coupled to the thiol group of CoA, releasing AMP and the acyl-CoA final products [6].

ACSL belongs to the AMP binding protein (AMPBP) super family, which occupy a critical position in the pathways of biosynthetic and biodegradable fatty acid-derived molecules [4]. According to the Commission on Enzymes of the International Union of Biochemistry with the diverse roles of acyl-CoA synthetase (ACS) in cell metabolism, ACS is classified by special catalyzing substrates, such as short- (C6–C8), medium- (C10–C12), long- (C14–C20), and very long- (>C22) chain fatty acids [7].

In higher plants, the importance of ACSL has been intensively studied. Nine ACSL genes have been cloned from *Arabiodopsis thaliana*, and most of ACSL enzymes display high activity levels with the fatty acids that make up the common structural and storage lipids in *Arabidopsis* tissues [8]. AtLACS1 and AtLACS2 have overlapping functions in both wax and cutin synthesis [9],[10]. AtLACS1, AtLACS2, and AtLACS3 can facilitate fatty acid uptake in yeast and have the dual functionality of yeast and mammalian fatty acid transport protein (FATP) enzymes [11]. AtLACS1 and AtLACS4 with combined activity are required for proper pollen coat formation [12]. AtLACS6 and AtLACS7 coding for peroxisomal ACSL proteins are involved in peroxisomal fatty acid β-oxidation [13], [14]. AtLACS9, a major chloroplast ACSL localized in the plastid envelope, has overlapping functions with AtLACS1 in seed oil biosynthesis [15], [16]. *BnACS6* cloned from *Brassica napus,* is highly expressed in lipogenic tissues, which might be involved in the lipid synthesis [17]. *GhACS1* from *Gossypium hirsutum* is essential for normal microsporogenesis in early anther development of cotton [18]. Additionally, putative ACSL enzymes are found in *Ricinus communis*, *Aegilops tauschii* and *Triticum urartu* [19], [20], [21].

However, despite the substantial information on peroxisomal metabolic pathways and the identification of many ACSL enzymes, little is known about the soybean ACSL enzymes until now. Soybean is one of the most important oil crops, providing about 30% of the edible oil in the world. The growing demand for vegetable oil has focused research toward on the metabolism of lipids in soybean [22]. So we cloned *GmLACS* from *Glycine max* previously, which may relate to the lipids synthesis [23]. In the present study, we identified another soybean ACSL, called *GmACSL2*. This gene resides at peroxisome, highly expresses after the first day germination, enhances the β-oxidation efficiency in yeast and reduces the content of fatty acids and lipids in yeast and hairy roots. Results of this study suggest that peroxisomal GmACSL2 probably be involved in the storage lipid degradation during seed germination.

## Methods

### Plant materials and growth conditions

Soybean (*G. max* cv. Williams 79) seeds were surface-sterilized as previously described [24]. For cloning and expression analysis of *GmACSL2*, plants were germinated and grown at 28°C under 12 h light/12 h dark photoperiod in a green house. For hairy root induction, the sterilized seeds were germinated on solid B5 medium (Phytotechnology laboratories, America). Cotyledonary node were cut from sterile seedlings as explants for transformation as previously described [25]. For germination experiment, soybean seeds were soaked in moist rock fiber in the dark for 6 days [26].

### Sequence analysis

Based on the homology cloning, gene searching was performed using tblastn in the NCBI database (http://www.ncbi.nlm.nih.gov/). The NCBI open reading frame (ORF) finder was used to find the ORF of the sequence. Domain prediction was done at ExPASy Proteomics Server (http://us.expasy.org/prosite/) and Genedoc [27]. A phylogenetic tree was constructed and exhibited using MEGA4 [28]. The website (http://www.nii.res.in/pred_acs_substr.html) was used to predicate ACS classification. Membrane association domain analysis was performed on the website (http://www.ch.embnet.org/software/TMPRED_form.html).

### Cloning and vector construction

RNA samples were isolated from the leaves of 5-day-old seedlings using plant Trizol reagent (Vigorous Biotechnology, China). 2 µg aliquot of total RNA was used for cDNA synthesis by M-MLV Reverse Transcriptase (TAKARA, Japan). The ORF primers of GmACSL2 are: GmACSL2-1F: 5′-ATGGCGACAATTCCTATCACCTAC-3′ and GmACSL2-1R: 5′ -TTACATGTATAGATTGTCTATTTGCTCCC-3′. The primers were designed by Primer Premier 5.0 [29]. The PCR conditions were as follows: 95°C for 5 min; 35 cycles of 95°C for 40 s, 58°C for 40 s, and 72°C for 2 min; and an additional step of 72°C for 10 min. The amplified products were cloned into pMD18-T vector (TAKARA, Japan) and then sequenced.

The PCR product of *GmACSL2* from pMD18-T Vector was digested with *BamH*I/*Xba*l, and then subcloned into vector pYES2 (Invitrogen, America) to generate the pYES2-GmACSL2 plasmid for yeast vector construction. *GmACSL2* was first subcloned into the pENTR vector for subcellular localization, and then LR recombined with pK7FWG2.0 to obtain pK7FWG-GmACSL2-eGFP through gateway system according to the protocol of Invitrogen [30]. The Plasmids of pCXDR-SSE1-dsRed were constructed following the method described by Chen et al [31]. *GmACSL2* was subcloned into the vector pGFPGUSPlus (pCAMBIAl305.1113) for soybean hairy root induction, with the *HPTII* gene as the selective marker and GUS/GFP as the reporter gene [32].

### Yeast complementation test

The plasmids of pYES2-GmACSL2 and empty vector pYES2 were transformed into *Saccharomyces cerevisiae* YB525 strain (ATCC), and the yeast complementary test was performed as previously described [33]. After 3 days of culture, clones were picked from selected drop-out uracil solid medium containing 2% yeast nitrogen base (Sangon Biotech, China), 0.077% complete supplement mixture without uracil (Shanghai Genomics, China), 2% dextrose, and 2% agar. Representative colonies were further identified by PCR. The positive yeast cells were washed twice by 2 M sorbitol and cultured in drop-out uracil liquid medium containing 2% galactose without dextrose to induce the *GmACSL2* expression. After 4 h induction, 10 µl of induced cultures were pipetted into 3 ml drop-out uracil liquid medium containing 98 µM fatty acid (12∶0, 14∶0, 16∶0, 18∶0, 18∶1, and 22∶1) as carbon source and then incubated at 30°C for 5 days. The growth rates of the yeast cells were determined with a spectrophotometer (Eppendorf Biophotometer, Germany).

### Assay for ACS Activity

The ACS assays were performed as previously described with minor modification [8]. Yeast cells carrying the indicated plasmids were harvested after galactose induction. 1-[^14^C] oleic acid was used as a substrate. The reaction mixture (100 µl) consisted of 100 mM bis-tris-propane (pH 7.6), 10 mM MgCl_2_, 5 mM ATP, 1 mM CoA, 2.5 mM dithiothreitol, 0.1% bovine serum albumin (BSA), 20 µM 1-[^14^C] oleic acid and 20 µg yeast protein. The reaction was incubated at 25°C for 20 min, stopped by addition of 100 µl of 10% (v/v) acetic acid in isopropanol, and extracted twice with 900 µl of hexane saturated with 50% (v/v) isopropanol. Enzyme activity was determined by measuring the counts in the aqueous phase using a liquid scintillation counter. All experiments were repeated 3 times.

### Subcellular localization

Both plasmids of pK7FWG-GmACSL2-eGFP and pCXDR-SSE1-dsRFP were transformed into *Agrobacterium GV3101*. After overnight culture, the transformed strains were resuspended with 10 mM MgCl_2_ (OD = 0.8) and then injected into *Nicotiana benthamiana* leaves as previously described [34]. Then the tobacco leaves were observed under a confocal fluorescence microscope (TCS SP5, Leica). Excitation and emission filters Ex450 nm to 490 nm/BA520 nm to 560 nm were used for eGFP and Ex540 nm to 580 nm/BA600 nm to 660 nm for dsRFP.

### Real-time PCR

Total RNA from different soybean tissues, germinating seedlings and developing seeds was extracted, and 200 ng RNA was used for cDNA synthesis (SYBR Prime Script RT-PCR Kit, TaKaRa, Japan). *Actin* (actin-F: 5′-GAGCTATGAATTGCCTGATGG-3′ and actin-R: 5′- CGTTTCATGAATTCCAGTAGC -3′) with an amplification length of 188 bp was used as the internal control [35]. The primer pair of *GmACSL2* was GmACSL2-2F: 5′- CTTCCTTTCACCCTGCAATTTC -3′ and GmACSL2-2R: 5′- TTCCACTCATCTGAGGCCACAC -3′ with an amplification length of 204 bp. These primers were designed in the nonconservative regions. Real-time PCR was preformed using ABI PRISM 7300 real-time cycler (Applied Biosystems). Melting curves were constructed using Dissociation Curves software to ensure that only a single product was amplified. According to Livak and Schmittgen’s method [36], the relative expression intensity was based on 2^−ΔΔCT^ equation, which was calculated by the internal software of ABI PRISM 7300 according to 2^−(ΔCT GmACSL2-ΔCT actin)^.

### Expression of GmACSL2 in S.*cerevisiae pep4*

The recombinant plasmids of pYES2-GmACSL2 and pYES2 were transformed into *S.cerevisiae pep4* cells. The positive clones were cultured in YPD liquid medium (2% bacteriological peptone and 1% yeast extract) supplemented with 2% dextrose**.** The yeast cells were washed twice with 2 M sorbitol and then transferred to YPD liquid medium supplemented with containing 2% galactose to induce the *GmACSL2* expression for 12 h. For RT-PCR assays of different transformed lines, the primer of *GmACSL2* was GmACSL2-3F (5′-GGCGACAATTCCTATCACCTAC-3′) and GmACSL2-3R (5′- GACCT TGAGCCCCTTACCACAT-3′). *Actin* (actin-2F 5′- ATGGATTCTGGTATGTT CTA-3′ and actin-2R 5′- GATACCTCTCTTGGATTGAGC-3′) was used as an internal control. Subsequently, 5 µl products of each reaction were electrophoresed on 1% agarose gel stained with SYBR Green.

### β-oxidation assay

β-oxidation assay in *pep4* cells were performed as previously described [37]. Cells were grown overnight in media containing oleate to induce fatty acid β-oxidation. The substrates used were 1-[^14^C] oleic acid. β-oxidation measurements in intact cells were followed by quantification of [^14^C] CO_2_ and ^14^C-labelled β-oxidation products in a liquid scintillation counter. The β-oxidation activity in wild-type cells in each experiment was taken as reference (100%).

### Induction of soybean hairy root

pGFPGUS and pGFPGUS-GmACSL2 plasmids were transformed into *Agrobacterium rhizogenes* K599. Induction of hairy root was conducted according to the method described previously with some modifications [38]. Cotyledonary node explants from 5 d seedlings were harvested, wounded with a scalpel, dipped into K599 for half an hour, and then incubated on MCC medium covered with a sterile filter paper at 23°C under illumination for 5 d. The MCC medium consisted of 1/10 MS, 3% sucrose, 3.9 g/L MES, 150 mg/L cysteine, 150 mg/L dithiothreitol, and 20 mg/L acetosyringone. After washing with 1/2 MS lipid medium, the explants were cultured on 1/2 MS medium supplied with 3% sucrose and 0.6 g/L MES, carbenicillin disodium and *hygromycin* B at 25°C under illumination. After 10 d to 14 d induction, hairy roots that formed at the wounded sites were tested for GUS activity and GFP fluorescence detection. The hairy roots were cut from explants in solid 1/2 MS medium. After identifying, the transgenic hairy roots and the control were propagated in 1/2 MS lipid medium for two weeks until analysis.

### Lipids and fatty acids content analyses

For lipids content analysis, yeast cells were collected and stained with Sudan Black B as previously described [39]. The absorbance was measured at 580 nm using unstained cells as the control. The hairy roots were lyophilized and harvest after propagating. The lipids of each dry hairy root lines (200 mg) were extracted using a Soxhlet Extractor (Buchi, Swiss).

For fatty acids content analysis, dry yeast cells (100 mg) and fresh hairy roots (500 mg) were collected and pulverized. Fatty acid extraction and measurement were carried out according to the method described by Larson et al. with some modifications [40]. Each vial was added with 500 µl 1 N HCl in methanol, 200 µl hexane and 10 µg heptadecanoic acids (Sigma) as an internal standard. The sealed vials were heated at 85°C for 2 h and cooled at room temperature. The hexane phase containing the fatty acid methyl esters partitioned from the aqueous phase by the addition of 250 µl 0.9% KCl. The hexane phase was transferred to another vial, and 1 µl aliquots were analyzed by GC 8000 (Thermo quest Separation Products, UK). Injections were made into a 30 m long 0.25 mm ID SGEBPX70 column (SGE, UK) using He as a carrier gas at 1 mL min^−1^ with a 30:1 split ratio. The oven was run isothermally at 110°C for 1 min and ramped to 180°C at 20°C min^−1^ then to 221°C at 2.5°C min^−1^.

### Statistical analysis

All data are presented as mean ± standard error of the mean (SEM). Student *t*-test was used to determine significance between individual comparisons. One-way ANOVA tests with Bonferroni’s corrections were used for multiple comparisons. The calculations were performed with SPSS version 11.0 statistical software. *P*<0.05 was considered statistically significant.

## Results

### Identification and cloning of *GmACSL2* in *G max*

*Arabidopsis* AtLACS1 to AtLACS9 (AAM28868 to AAM28876) were used as queries to tblastn *G. max* database, and two full-length cDNAs (AK245419 and AK245622) with high sequence similarity were found. One cDNA (AK245419), namely *GmLACS*, has been studied preliminarily [23]. Another cDNA (AK245622), namely *GmACSL2* according to the nomenclature for ACS enzymes [41], was selected. It shares 78% high identity with putative *RcACSL* cDNAs (XM_002520572 and XM_002520569). The full-length cDNA of *GmACSL2* was obtained by RT-PCR. Sequence analysis indicated that the full-length cDNA of *GmACSL2* is 2,250 bp with 97 bp 5′-untranslated regions, a predicted 2202 bp ORF, and 251 bp 3′-untranslated regions. The ORF of *GmACSL2* was predicted to encode a protein of 733 amino acid residues with a theoretical pI of 7.11 and a calculated molecular weight of 82.09 KDa.

**Figure 1:**
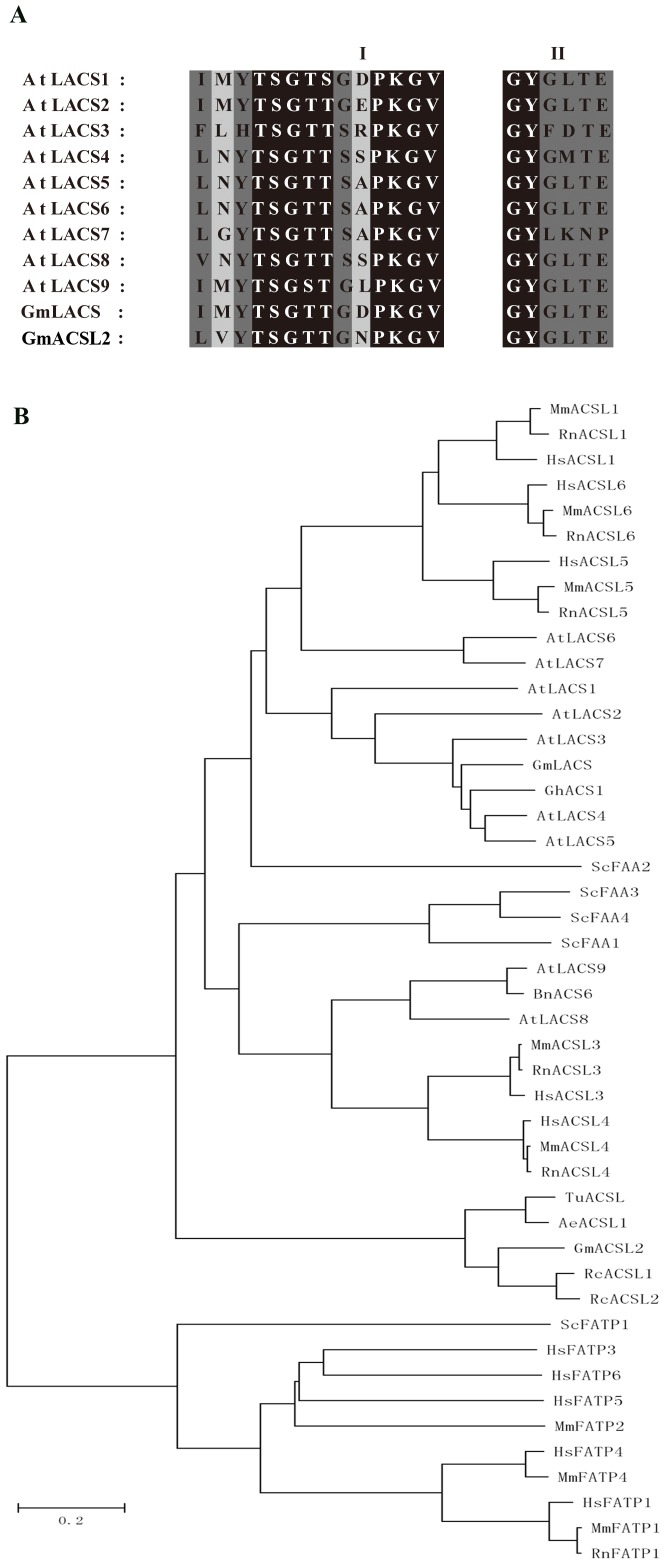
Sequence analysis of GmACSL2. (A) Multiple amino acid sequences alignment of *Glycine max* GmACSL2 and GmLACS sequence with *Arabidopsis thaliana* AtLACS1 to AtLACS9. The **I** and **II** blocks of shadows indicate the highly conserved acid residues in AMP-binding protein. Black frames indicate the higher conservation amino acids, dark grey frames indicate the conservation amino acids, and light frames indicate the lower conservation amino acids. The protein follows: AtLACS1 (AAM28868), AtLACS2 (AAM288689), AtLACS3 (AAM28870), AtLACS4 (AAM28871), AtLACS5 (AAM28872), AtLACS6 (AAM28873), AtLACS7 (AAM28874), AtLACS8 (AAM28875), and AtLACS9 (AAM28876). (B) Phylogenic analysis between GmACSL2 with other ACSL enzymes and FATP proteins from plant, mammalian and yeast. The ACSL enzymes include *G.max* GmLACS, *Arabidopsis thaliana* AtLACS1-9, *Gossypium hirsutum* GhACS1 (ABA00144), *Brassica napus* BnACS6 (CAC19877), *Ricinus communis* RcACSL1 (XP_002520618) and RcACSL2 (XP_002520615), *Aegilops tauschii* AeACSL1 (EMT11835), *Triticum urartu* TuACSL (EMS60031), *Homo sapiens* HsACSL1 (NP_001986), HsACSL3 (NP_004448), HsACSL4 (NP_004449), HsACSL5 (NP_057318), and HsACSL6 (NP_056071), *Mus musculus* MmACSL1 (NP_032007), MmACSL3 (XP_129894), MmACSL4 (NP_062350), MmACSL5 (AAH31544), and MmACSL6 (NP_659072), *Rattus norvegicus* RnACSL1 (NP_036952), RnACSL3 (NP_476448), RnACSL4 (NP_446075), RnACSL5 (NP_446059), and RnACSL6 (NP_570095), and *Saccharomyces cerevisiae* ScFAA1 (P30624), ScFAA2 (P39518), ScFAA3 (P39002), and ScFAA4 (P47912). FATP proteins include *Homo sapiens* HsFATP1 (NP_940982), HsFATP3 (NP_077306), HsFATP4 (Q6P1M0), HsFATP5 (Q9Y2P5), and HsFATP6 (NP_001017372), *Mus musculus* MmFATP1 (NP_036107), MmFATP2 (AAC40186), and MmFATP4 (XP_130079), *Rattus norvegicus* RnFATP1 (NP_036119), and *Saccharomyces cerevisiae* (EWH19453). The bars stand for evolutionary distance. Bar = 0.2.

Multiple sequence alignment of *Arabidopsis* AtLACS1 to AtLACS9, GmLACS, and GmACSL2 showed that AMP-binding domain signatures (PROSITE PS00455), [LIVMFY]-[E]-[VES]-[STG]-[STAG]-G-[ST]-[STEI]-[SG]-x-[PASLIVM]-[KR] are highly conserved in the members of the AMP-binding protein super family (Figure 1A) [8]. The sequence analyzed by ScanProsite Results Viewer also showed the notable AMP-binding domain in GmACSL2. There was 85.64% probability that GmACSL2 sequence was predicted to activate the long- chain fatty acids by a web-based prediction tool [7].

Phylogenetic analysis was conducted to visually compare the relationship of GmACSL2, other ACSL enzymes and FATP proteins include plant, yeast and mammalian (Figure 1B). The presence of genes on branches close to one another may represent certain levels of overlapping functions. The phylogenetic tree showed that GmACSL2 is more related to the ACSL enzymes and mainly formed a closely related cluster with the *R. communis* RcACSL1 and RcACSL2, *A. tauschii* AeACSL1 and *T. urartu* TuACSL, while GmLACS make a branch with GhACS1, AtLACS4 and AtLACS5. GmLACS and GmACSL2 probably are involved in the different lipid metabolism according to the presence on different branch. Membrane association domain analysis showed that there are 7 inside to outside helices and 6 outside to inside helices in GmACSL2 sequence. All these results above on predicted that the obtained *GmACSL2* is a putative ACS gene.

### Complementation of yeast mutant YB525

**Figure 2:**
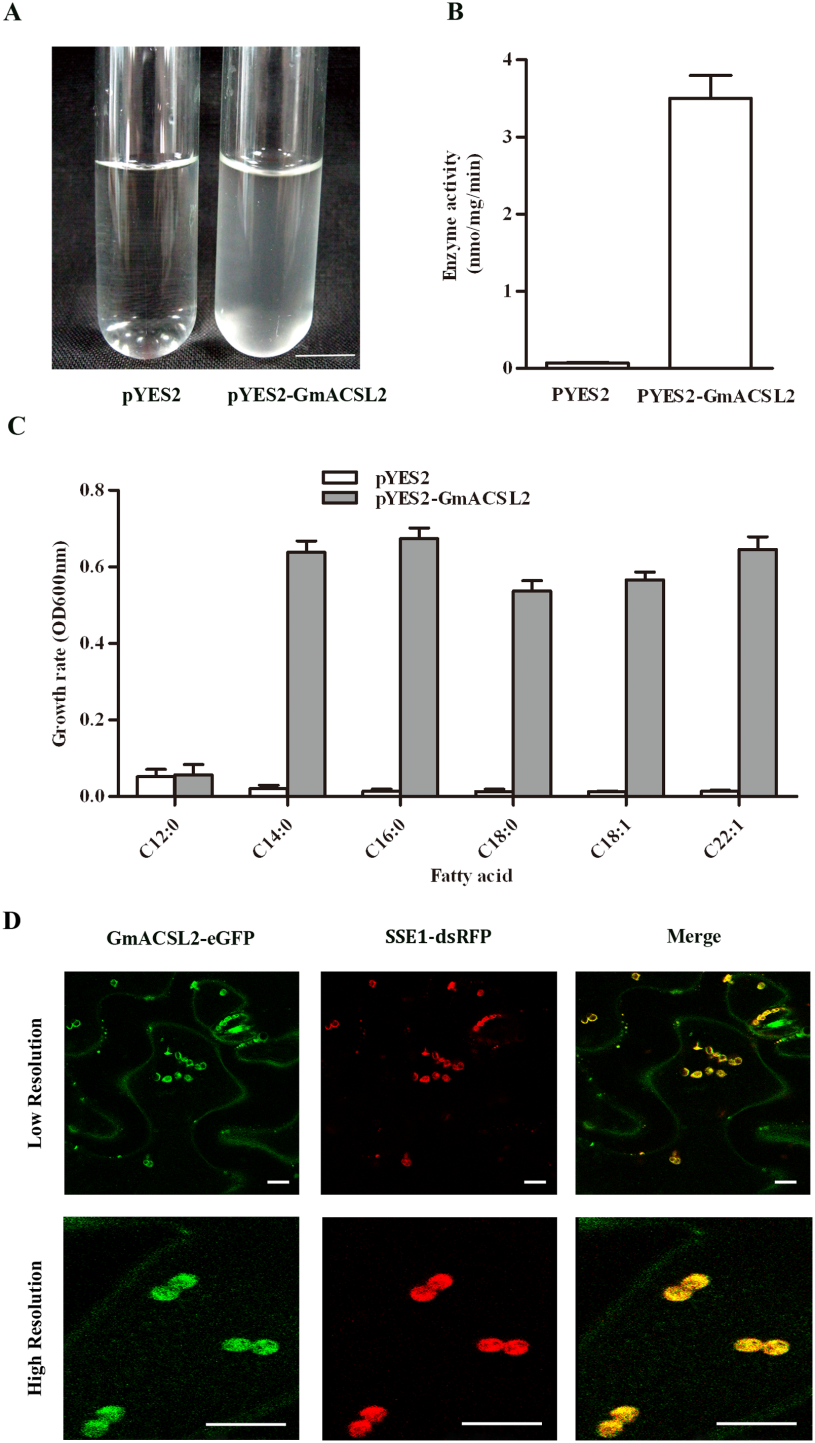
Yeast complementation test and subcellular localization. (A) Yeast complementation test. Right: culture of yeast strain YB525 cells containing the pYES2 empty plasmids; Left: culture of yeast strain YB525 cells containing the pYES2-GmACSL2 plasmids. Bar = 1 cm. (B) ACS enzyme activities. YB525 cells carrying the pYES2 or pYES2-GmACSL2 plasmids were harvested after galactose induction for 18 h. 1-[^14^C] oleic acid was used as a substrate. Enzyme activities were measured based on the [^14^C] label incorporated into the acyl-CoA fraction per assay. Values are means of triplicate with standard deviation (SD). (C) Growth rate of the transformed line in different fatty acid culture medium. YB525 was transformed with the pYES2-GmACSL2 and empty PYES2 plasmids, which were cultured in liquid medium with various fatty acids (12∶0, 14∶0, 16∶0, 18∶0, 18∶1, and 22∶1) as the sole carbon source. (D) Subcellular localization. Fluorescence signals of eGFP were detected in cells expressing GmACSL2-eGFP fusion protein by Leica TCS scanning confocal microscope (left panels). Fluorescence signals of dsRFP were detected in cells expressing SSE1-dsRFP fusion protein (middle panels). Right panels were merged by left and middle panels. The upper panels are low-resolution pictures and the lower panels were high-resolution pictures. The immunofluorescence was done with tobacco leafs. Bar = 10 µm. Data are presented as the mean ± SEM of three experiments.

GmACSL2 was subcloned into pYES2 and transformed into YB525 to determine whether the cloned *GmACSL2* cDNA encodes acyl-CoA synthetase enzyme capable of activating the fatty acids. This strain’s fatty acid activation (FAA) 1–4 genes were disrupted and it lacks activities of acyl-CoA synthetases to activate exogenous fatty acids [42]. Complementary test showed that the yeast cells transformed with pYES2-GmACSL2 restored ACS activity of YB525 compared with the control cells transformed with pYES2, which was not grown in medium containing fatty acids as the sole carbon source (Figure 2A). The lysates from cells after galactose induction for 18 h were used as enzyme sources for ACS activity assays. Lysates with GmACSL2 were enzymatically active, compared to lysates from control cells with an empty pYES2 vector (Figure. 2B). Then the transformants were cultured in liquid medium with various fatty acids, including lauric acid (12∶0), myristic acid (14∶0), palmitic acid (16∶0), stearic acid (18∶0), oleic acid (18∶1), and erucic acid (22∶1). YB525 transformed with pYES2-GmACSL2 showed normal growth in the medium containing 14∶0, 16∶0, 18∶0, 18∶1 and 22∶1 fatty acids, except for 12∶0 short-chain fatty acid (Figure 2C). These results indicated that *GmACSL2* encodes active acyl-CoA synthetase and preferably utilize long-chain, not the short chain fatty acids as the substrates.

### Subcellular localization of GmACSL2

The SSE1 protein, which localizes in the peroxisome, was selected as a maker to elucidate the subcellular localization of GmACSL2 [43]. The GmACSL2 was fused with eGFP (green fluorescent protein) to construct the pK7FWG-GmACSL2-eGFP plasmids, and SSE1 was fused with the dsRFP (red fluorescent protein) to construct pCXDR-SSE1-dsRFP plasmids. Both of the plasmids were injected into tobacco leaves. Tobacco cells that expressed GmACSL2-eGFP fusion protein (Figure 2C, left panel) and SSE1-dsRFP fusion protein (Figure 2C, middle panel) were examined under a Leica confocal laser-scanning microscope. The localization of eGFP overlapped with that of dsRFP (Figure 2C, right panel). Similar to SSE1, the GmACSL2 protein is chiefly localized in the peroxisome. Peroxisomes are membrane-bound organelles involved in the metabolism of fatty acids. Thus we predicted that peroxisomal GmACSL2 might participate in this process of fatty acids metabolism.

### Expression pattern of *GmACSL2* in different tissues and germinating seedlings

**Figure 3:**
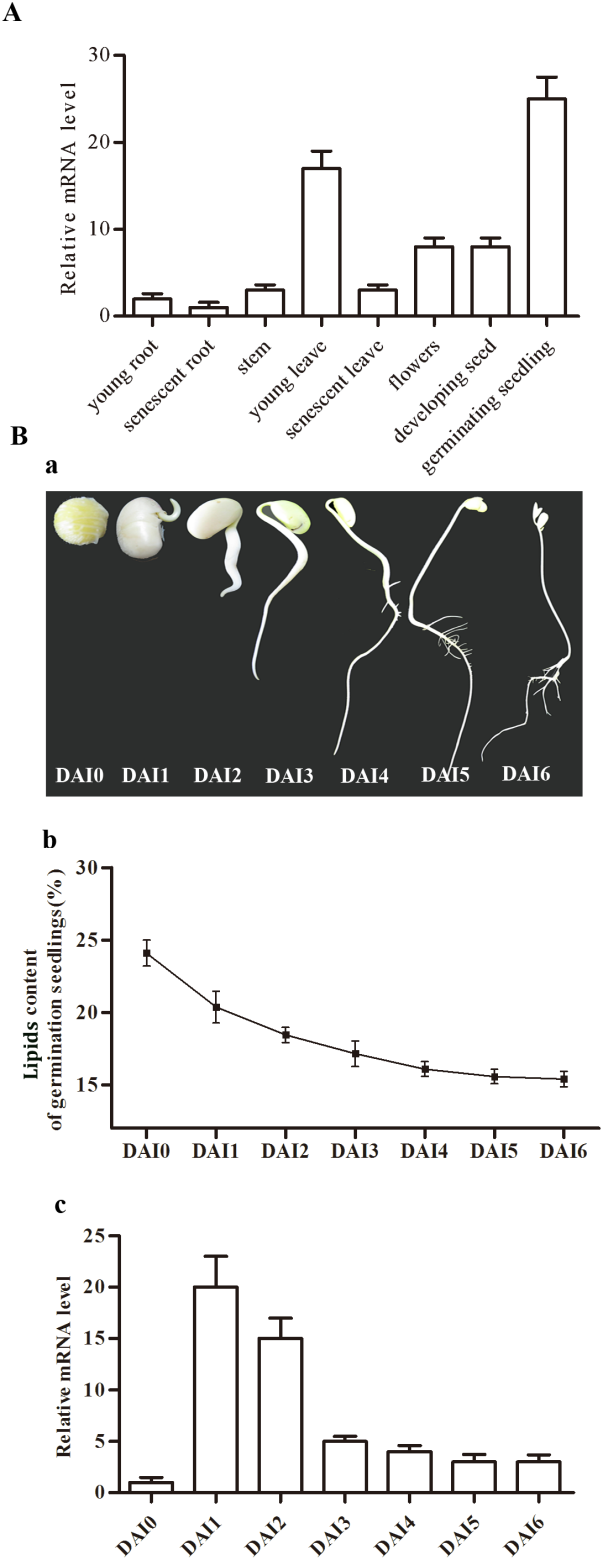
Real-time PCR analysis of expression of *GmACSL2* in soybean. (A) Expression pattern of *GmACSL2* in different tissues. Total RNA was extracted from young roots, senescent roots, stems, young leaves, senescent leaves, flowers, developing seeds, and germinating seedlings. The actin gene served as the positive control. (B) Expression pattern of *GmACSL2* in germination seedling under dark. (a) Germination seedlings from 0 day to 6 day after imbibition (DAI). (b) Lipids content of seedlings from 0 day to 6 day after imbibition. (c) Real-time analysis of the expression of *GmACSL2* from 0 day to 6 day after imbibition. Data are presented as the mean ± SEM of three experiments.

Real-time PCR was performed to investigate the expression pattern of *GmACSL2* in different tissues. *GmACSL2* was expressed in almost all tissues, particularly in young leaves and germinating seedlings (Figure 3A). The expression of *GmACSL2* was much higher in young tissues than senescent tissues. To assess the expression of *GmACSL2* in different stages of germinating seedlings, soybean seeds were germinated under dark for 7 days. During seeds germination 0–7 day after imbibition (DAI) under dark (Figure 3B-a), the oil contents of the seedlings were gradually decreased (Figure 3B-b). The expression levels of *GmACSL2* increased to a sharp maximum at 1 and 2 DAI and during postgerminative growth, the expression levels of *GmACSL2* gradually declined (Figure 3B-c). Peroxisomal *GmACSL2* was highly expressed in germinating seedlings and highly increased after 1 day imbibition, suggesting its involvement in lipid metabolism during seed germination.

### Overexpression of *GmACSL2* in yeast mutant *pep4*

**Figure 4:**
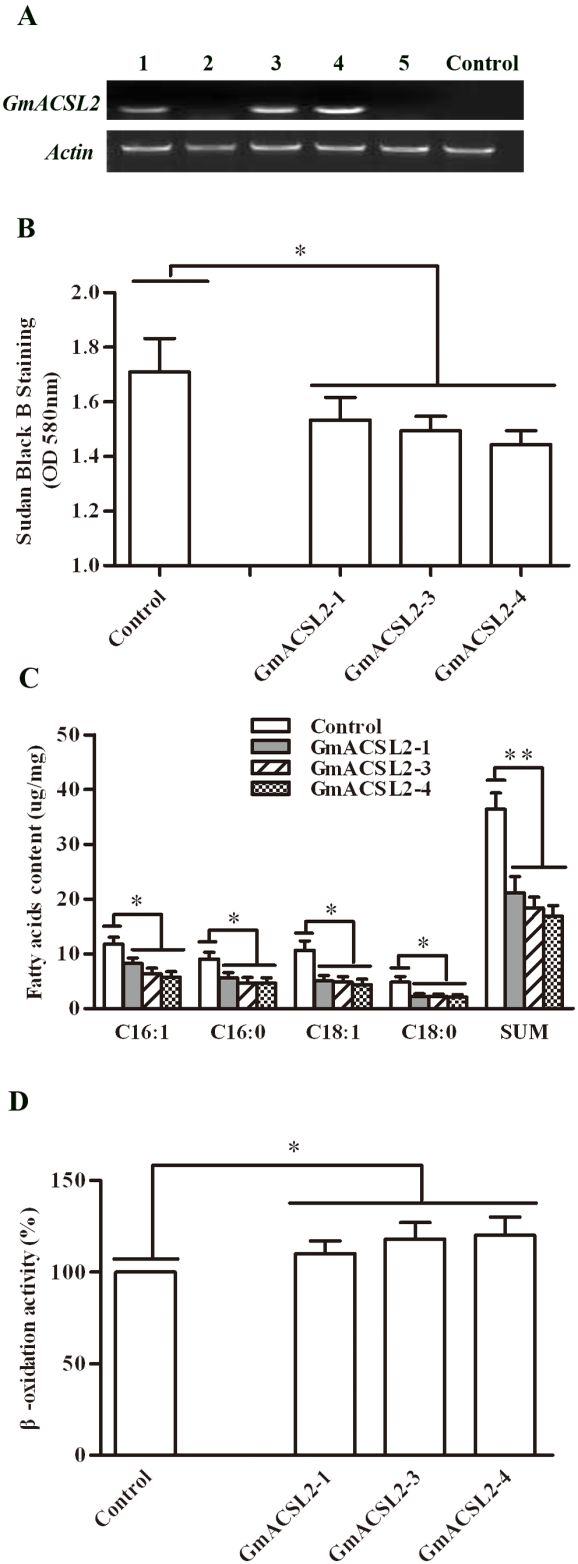
Overexpression of *GmACSL2* in yeast *pep4*. (A) Expression analysis of *GmACSL2* in different transformed lines by RT-PCR. Numbers 1 to 5 represents the five lines, GmACSL2-1, GmACSL2-2, GmACSL2-3, GmACSL2-4, and GmACSL2-5, transformed with pYES2-GmACSL2. The actin gene was used as the control for equal gel loading. Control represents the line transformed with pYES2 empty vector. (B) Sudan black B staining. The cells of three transformed lines GmACSL2-1, GmACSL2-3, and GmACSL2-4 were stained with Sudan Black B. The absorbance was measured at 580 nm and the line transformed with pYES2 vectors as control. (C) Fatty acids analysis comparisons between three transformed lines GmACSL2-1, GmACSL2-3 and GmACSL2-4 and the control line. The four major fatty acid species C16∶0, C16∶1, C18∶0, C18∶1 and the total fatty acids content in the yeast were detected by gas chromatography-mass spectrometry. (D) β-oxidation assay comparisons between three transformed lines GmACSL2-1, GmACSL2-3 and GmACSL2-4 and the control line. Oleate β-oxidation measurements in cells were followed by quantification of [^14^C] CO_2_ and ^14^C-labelled β-oxidation products in a liquid scintillation counter. The β-oxidation activity in control cells in each experiment was taken as reference (100%). Data are presented as the mean ± SEM of the three experiments. *p<0.05, **p<0.01.

The plasmids of pYES2-GmACSL2 were transformed into *S.cerevisiae pep4*, which lacks the expression the major hydrolase and accumulates the exogenous protein sustained expression without degradation [44]. RT-PCR results indicated that *GmACSL2* could be expressed in the GmACSL2-1, GmACSL2-3, and GmACSL2-4 transformed lines in *pep4* (Figure 4A). The expression of *GmACSL2* in the GmACSL2-3 and GmACSL2-4 was stronger than the GmACSL2-1 transformed line. The lipid bodies of yeast were prominent in the retardation phase of growth, and could be stained with Sudan Black B to indicate the relative content of cells. The staining results showed that the absorbance values of GmACSL2-1, GmACSL2-3, and GmACSL2-4 transformed lines decreased to 10.3%, 12.5% and 15.6% compared with the negative control, respectively (Figure 4B). Further we tested the four major fatty acid species, palmitic acid (C16∶0), palmitoleic acid (C16∶1), stearic acid (C18∶0), and oleic acid (C18∶1), and total amount in yeast by GC-MS. These fatty acids decreased to different levels in three of the pYES2-GmACSL2 transformed lines compared with the pYES2 control line and the total content of these fatty acids decreased by approximately 41%, 49%, and 53% in the GmACSL2-1, GmACSL2-3, and GmACSL2-4 transformed lines, respectively, compared with the negative control (Figure 4C). C16∶0, C16∶1, C18∶0, and C18∶1 decreased by 51%, 48%, 59%, and 57% in the GmACSL2-4 transformed line, respectively, compared with the control (Figure 4C). *GmACSL2* overexpression decreased the lipid and fatty acid contents of yeast. At last we measured the rate of β-oxidation in *pep4* transformed with pYES2 and pYES2-GmACSL2 plasmids. We found that the β-oxidation efficiency increased about 10% after overexpression GmACSL2 in *pep4* (Figure 4D). The activities of oleate-β-oxidation of GmACSL2-1, GmACSL2-3, and GmACSL2-4 transformed line was 10.8 nmol/h/mg, 10.9 nmol/h/mg, and 11.0 nmol/h/mg, respectively, comparing to control line 9.8 nmol/h/mg. *GmACSL2* overexpression enhances the efficiency of β-oxidation. Thus, GmACSL2 may be involved in the metabolism of lipid and fatty acid degradation.

### Overexpression of *GmACSL2* in soybean hairy root

**Figure 5:**
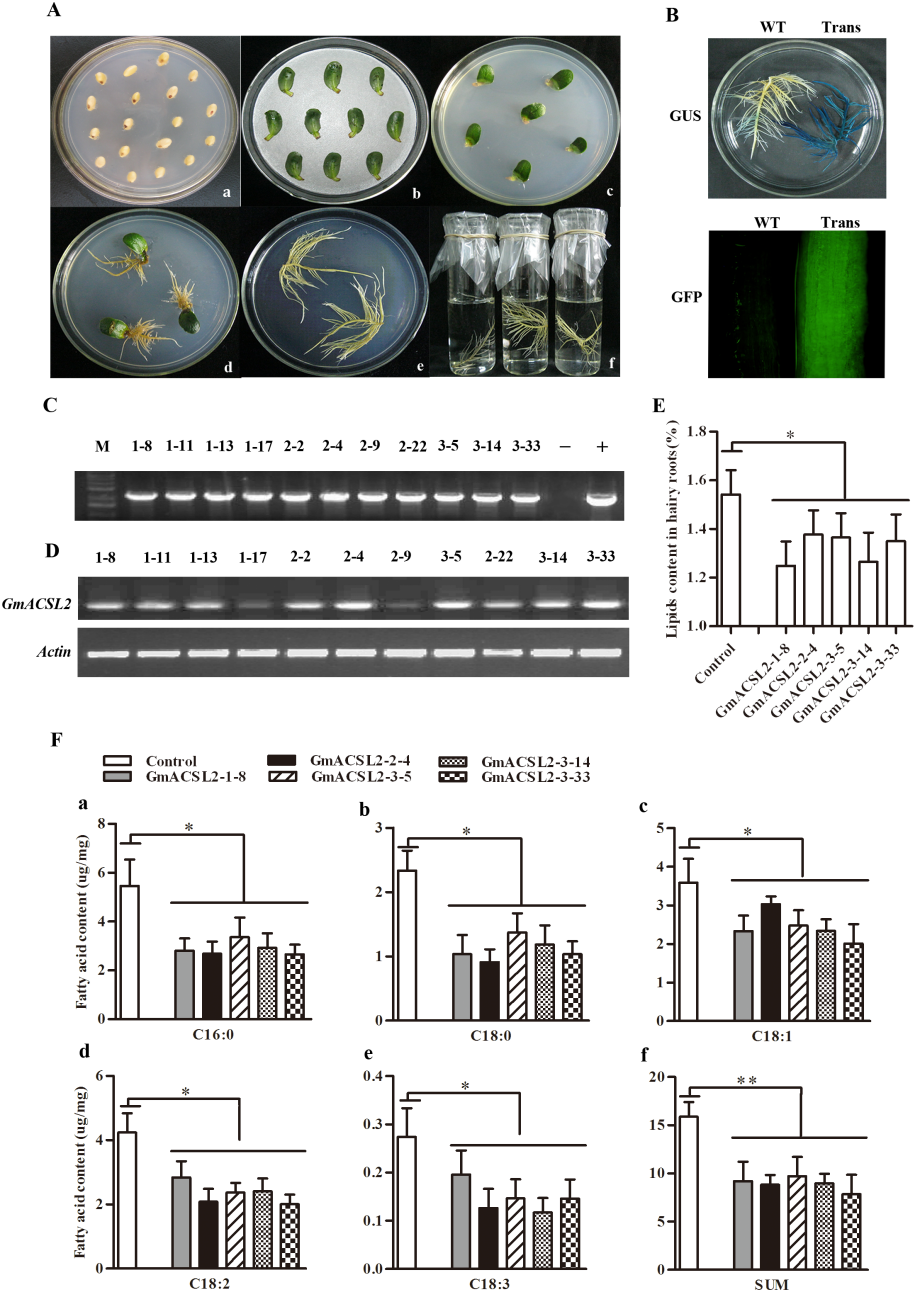
Overexpression of *GmACSL2* in transgenic hairy roots. (A) Hairy roots were induced by *A. rhizogenes.* (a) Soybean seeds were germinated in germination medium; (b) Explants were co-cultivated with *A. rhizogenes in* MCC medium; (c) Hairy roots were induced in 1/2 MS medium with hygromycin B as selection; (d) Hairy roots appeared at the wounding sites of the explants in 1/2 MS medium; (e) Hairy roots were grown in 1/2 MS solid medium; (f) Hairy roots were propagated in 1/2 MS liquid medium. (B) GUS histochemical staining and GFP fluorescence signal detection. After extended culture, the hairy roots were stained by GUS (upper panel) and signals of fluorescence GFP observation (lower panel). WT represents the wild-type soybean hairy roots hairy roots transformed with the K599 strain only; Trans represents the soybean hairy roots transformed with the control vector pGFPGUS and pGFPGUS-GmACSL2. (C) PCR analysis of hairy roots DNA using the primers to amplify the fragment of the GmACSL2 gene to further identify the transgenic line of GmACSL2. M, Marker; “+”, positive control, GmACSL2 plasmid was used as template; “−”, negative control, soybean hairy roots were transformed with the control vector pGFPGUS as template; 1–8, 1–11, 1–13, 1–17, 2–2, 2–4, 2–9, 2–22, 3–5, 3–14, and 3–33, individual lines transformed with the binary vector pGFPGUS-GmACSL2. (D) Expression analysis of *GmACSL2* in different transformed lines by RT-PCR. 1–8, 1–11, 1–13, 1–17, 2–2, 2–4, 2–9, 2–22, 3–5, 3–14, and 3–33 represent different transformed hairy roots overexpression with *GmACSL2*. Soybean actin gene was used as an internal control. (E) Analysis of lipid contents in the transgenic lines by soxhlet extraction. Control, transgenic hairy roots induced by pGFPGUS control vector; GmACSL2-1-8, GmACSL2-2-4, GmACSL2-3-5, GmACSL2-3-14, and GmACSL2-3-33, the five transgenic hairy root lines, induced by the pGFPGUS-GmACSL2 vector. (F) Fatty acid analysis comparisons between five *GmACSL2* transgenic lines and the control. The five major fatty acid species C16∶0, C18∶0, C18∶1, C18∶2, C18∶3 and the total fatty acids content in hairy roots were detected by gas chromatography-mass spectrometry. Data are the mean ± SEM of the three experiments. *p<0.05, **p<0.01.

The plasmids of pGFPGUSPlus-GmACSL2 were transformed into *A. rhizogenes* for soybean hairy root induction. After soybean seeds germination (Figure 5A-a), the explants were cut and co-cultivated with *A. rhizogenes* in MCC medium (Figure 5A-b) for 5 d, and then the explants were induced on 1/2 MS medium (Figure 5A-c). After 10 d to 14 d, hairy roots were formed at the wound site (Figure 5A-d). Then the hairy roots were cut from cotyledons and cultured in 1/2 solid medium before identity (Figure 5A-e). The transgenic hairy roots were identified by GUS activity staining (Figure 5B, upper panel), GFP fluorescent protein observation (Figure 5B, lower panel) and genomic PCR amplification (Figure 5C). At last eleven transformed lines were established and propagated in 1/2 MS liquid medium (Figure 5A-f). Five higher expression transformed lines of GmACSL2-1-8, GmACSL2-2-4, GmACSL2-3-5, GmACSL2-3-14, and GmACSL2-3-33 were selected by RT-PCR analysis of *GmACSL2* expression in different transformed hairy root lines (Figure 5D).

After 15 d of extended culture, lipids were extracted from the hairy roots through soxhlet extraction. *GmACSL2* overexpression significantly decreased the lipids contents by 18.9%, 10.7%, 11.5%, 17.9%, and 12.5% in the hairy roots transformed lines GmACSL2-1-8, GmACSL2-2-4, GmACSL2-3-5, GmACSL2-3-14 and GmACSL2-3-33, respectively, compared with the hairy roots transformed by the vector control (Figure 5E). The levels of five major fatty acid species C16∶0, C18∶0, C18∶1, C18∶2 and C18∶3 fatty acids were evaluated, compared with the control. The contents of C16∶0, C18∶0, C18∶1 C18∶2, and C18∶3 reduced by approximately 47%, 52%, 31%, 44%, and 46% in the five transgenic hairy roots lines compared with the control, respectively (Figure 5F-a∼e). The total contents of the five fatty acids decreased by 42%, 44%, 38%, 43%, and 50% in the GmACSL2-1-8, GmACSL2-2-4, GmACSL2-3-5, GmACSL2-3-14, and GmACSL2-3-33 transformed lines compared with the control, respectively (Figure 5F-f). Overexpression of *GmACSL2* in soybean hairy roots decreased lipid and fatty acid contents, suggesting that GmACSL2 is an important enzyme that catalyzes the five fatty acids to form acyl-coenzymes involved in the metabolism of lipid degradation.

## Discussion

A growing body of evidence indicates that ACSL is important in lipids metabolism. This important class of enzyme factors is prominently involved in several fatty acid-derived metabolic pathways, including phospholipid, triacylglycerol, and jasmonate biosynthesis and fatty acid β-oxidation. In higher plants, many ACSL genes were cloned. However, little is known about the ACSL enzymes in soybean to date. The objective of this study was to identify ACSL proteins involved in soybean lipid metabolism. Through tblastn in soybean database, we identified and cloned two cDNAs (*GmLACS* and *GmACSL2*) from soybean. Sequence analysis showed a high degree of homology between GmACSL2/GmLACS and other ACSL enzymes. Expression pattern analysis of *GmACSL2* in different tissues showed that it is highly expressed in germinating seedling (Figure 3A). Thus, we focused on the GmACSL2.

Yeast complementation test demonstrated that GmACSL2 can encode active long acyl-CoA synthetase and capable of compensation yeast mutant YB525. This result is in accordance with the similar studies on AtLACSs from *Arabidopsis* [8]. The transformation of *GmACSL2* into YB525 not only provides an efficient way to testify the activity of the enzyme but also gives a way to analyze the preferred substrate of GmACSL2. When the transformed yeasts were survived in auxotroph medium containing different fatty acids from long-chain and short-chain fatty acids, we found that the yeast transformed with GmACSL2 preferring to make use of the long chain fatty acids (C14 to C22) over short-chain (C12). These results indicate that GmACSL2 displays high ACSL activity in catalyzing the conversion of long-chain fatty acids to acyl-CoAs.

The localization of plant ACSL proteins in different organelle fractions is related to their functions [15], [17], [18]. An earlier study revealed that AtLACS6 and AtLACS7 proteins are localized in peroxisomes and involved in fatty acid β-oxidation during the seed germination [4], [13]. The bioinformatics analysis of GmACSL2 showed no obvious localization sequences like AtLACS6 and AtLACS7. However, co-expression assay showed that GmACSL2 mainly localizes in the peroxisome (Figure 2C). Maybe the sequence in GmACSL2 is not obvious to find. The localization of GmACSL2 was done in tobacco leafs, and not in soybean. We find that tobacco leaves can rapidly and dependably show the protein location. This transient expression of vectors was highly efficient comparing to soybean leaf is difficult for transfection. Some structures marked by GmACSL2-GFP are not peroxisomes, maybe is other locations like cytoplasm.

In germinating seedlings, a tight correlation exists between the timing of peroxisomal ACSL expression and the utilization of stored fatty acids [5]. Peroxisomal *GmACSL2* appears to be highly expressed in young leaves and germinating seedlings. We checked the expression profiles of *GmACSL2* during germination under dark, wherein the seed must depend on the storage oil for survival, avoiding the effect of photosynthesis. After 7 day, the seedling began to decay (data is not shown). The expression profile of *GmACSL2* peaked at 1 and 2 DAI (Figure 3B), mirroring the expression profiles of AtLACS6 and AtLACS7, which involved in β-oxidation or the glyoxylate cycle. Thus, we proposed that *GmACSL2* might be related to storage lipid metabolism in peroxisome during seed germination.

*GmACSL2* overexpression in *pep4* reduced the content of lipids and the important composition of fatty acids compared with the control. The content of lipids and fatty acids decreased as the expression level of *GmACSL2* increased. Therefore, *GmACSL2* overexpression in yeast correlates with the decreased levels of TAGs. We also found that the β-oxidation efficiency is increased about 10% after overexpression *GmACSL2* in *pep4*. It is clear that GmACSL2 indeed increases selectively the β-oxidation of lipids. In complementation test, the GmACSL2 still can rescue a growth defect in yeast YB525, although lots of lipids (membranes) are needed for the new cells. In the yeast, these are storage lipids accumulate during stationary growth phase within organelles known as lipid bodies. The storage lipid synthesis is non-essential and there are many lipids bodies in yeast [45]. We speculate that the cell may own a clever mechanism for survival and reduce the lipid bodies’ TAGs, not the member lipids to enhance β-oxidation efficiency. Meanwhile, we also overexpressed *GmACSL2* in soybean hairy roots. Compared with the yeast system, the hairy root system is more complicated. We selected the higher expression transgenic lines to evaluate the content of lipids and fatty acids because of the basic level expression of *GmACSL2* in control hairy roots. The content of lipids and the important composition of fatty acids were also reduced compared with the control. Thus, *GmACSL2* overexpression in *pep4* and hairy root can enhance the efficiency of lipid degradation and the activation of long fatty acids transported into the peroxisome. During degradation, TAG was released to many of the activated fatty acids to involve in the β-oxidation pathway for energy supply.

In *S.cerevisiae* and plants, β-oxidation occurs exclusively in peroxisomes. Fatty acids can be imported into the organelle through two independent mechanisms. In yeast, medium-chain fatty acids are predominantly imported as free fatty acids to be activated inside the peroxisome by acyl-CoA synthetase Faa2p. By contrast, long-chain fatty acids are activated predominantly in the cytoplasm and transported as CoA esters across the peroxisomal membrane [46]. To enable the β-oxidation of external activated fatty acids, a system in soybean similar to yeast would be necessary where medium-chain fatty acids are activated inside the peroxisomal membrane while long-chain fatty acids are activated outside the peroxisomes and imported as acyl-CoAs. Based on the structure analyzed by Hisanaga et al [47], fatty acyl-CoAs are released into the cytosol. It may be suggested that the acyl-CoA synthetase encoded ACSL enzymes possibly located on the outer surface of the peroxisomal membrane, and supply acyl-CoAs to the FATP transporter [5]. Then the fatty acyl-CoAs probable finds their way preferentially to the inside of peroxisomes. In *Arabidopsis*, a protein with significant similarities to this transport system is involved in peroxisomal fatty acid β-oxidation [48]. Additionally AtLACS1, AtLACS2 and AtLACS3 possess the functions of fatty acid transport protein (FATP) enzyme [11]. The available evidence prompts that the GmACSL2 sequence may have membrane association domain and localize in peroxisomes, but the phylogenic analysis shows that the GmACSL2 is not belong to FATP protein family. According to the expression and localization of GmACSL2, we predicted that GmACSL2 probably have the similar function to activate the fatty acids, and supply acyl-CoAs to the FATP transporters through the peroxisomal membrane into the matrix of this organelle during seedling germination for β-oxidation.

Expression pattern analysis of *GmACSL2* in different tissues indicated this gene is also expressed in flower and developing seed. During oil seed maturation, parts of lipid are degraded; therefore, the final seed oil contents are not at the peak [49]. In this stage, regulation the genes involved in lipid degradation can be regulated to improve the oil content. In *Arabidopsis* seeds, suppression of the seed fatty acid reducer (SFAR) genes increases the contents of fatty acids [50]. In *B. napus*, the result showed that suppression of the triacylglycerol lipase gene during seed development results in up to an 8% gain in oil yield on either a seed, plant, or unit area basis in the greenhouse, with very minimal adverse effect on seed vigor [51]. Therefore, the GmACSL2 could be a promising target gene for suppression to improve oil yield in *G. max*.

In conclusion, we identified a peroxisomal ACSL from soybean. The expression pattern of this gene is strongly induced during germination. *GmACSL2* overexpression in yeast and hairy root can reduce the contents of lipids and fatty acids. All these results suggest that GmACSL2 encoding ACSL activity involved in the lipids degradation pathway during the seed germination.
